# Nutraceuticals and Metastasis Development

**DOI:** 10.3390/molecules25092222

**Published:** 2020-05-08

**Authors:** Lara Saftić Martinović, Željka Peršurić, Krešimir Pavelić

**Affiliations:** 1 University of Rijeka, Department of Biotechnology, Radmile Matejčić 2, HR-51000 Rijeka, Croatia; lara.saftic@biotech.uniri.hr (L.S.M.); zpersuric@biotech.uniri.hr (Ž.P.); 2 Juraj Dobrila University of Pula, Faculty of Medicine, HR-52100 Pula, Croatia

**Keywords:** nutrigenomics, metastases, small molecules, antioxidants, high-fat diet, phytoestrogens

## Abstract

Nutrigenomics is a discipline that studies the effects of various dietary components on gene expression and molecular mechanisms via “omics” technologies. Many studies are focused on revealing the pathways of the anticancer properties of various nutraceuticals. However, it has been shown that metastasis, a multifactorial disease that develops from primary tumors in cascades, is responsible for almost 90% of cancer deaths. Regrettably, the effects of consumption of different nutraceuticals on metastasis development have not yet been sufficiently explored. A few studies on the subject have revealed the promotional effects of some nutraceuticals on metastasis development. Additionally, it has been shown that certain compounds can have beneficial effects on reduction of the primary tumor, but afterwards promote the spread of metastases. Therefore, in this review we discuss results published in the past five years focused on the effects of different nutraceuticals on metastasis development.

## 1. Introduction

**Figure 1:**
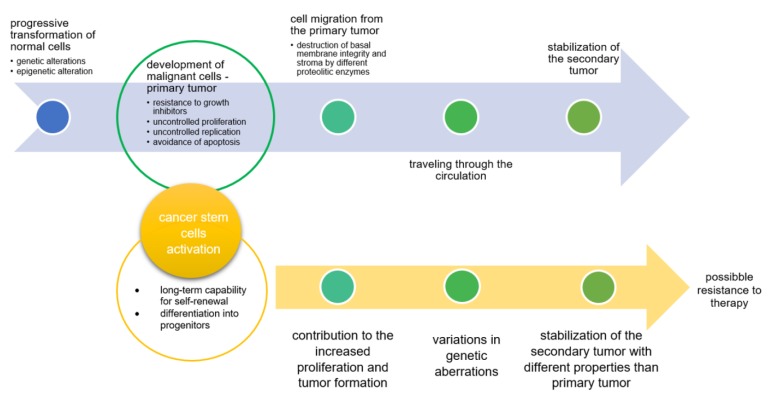
Steps in tumor progression [2].

After cancer development at the primary site due to uncontrolled accumulation of genetic and epigenetic alterations that lead to the progressive transformation of normal cells into malignant cells, the second step often includes metastasis formation (Figure 1). It is estimated that among all cancer deaths, metastasis is responsible for almost 90%. Cancer metastasis represents a multifactorial illness that develops in cascades. The first step comprises cancer cell proliferation at the primary site of the tumor, after which proliferated cancer cells migrate from primary site and invade the basement membrane and the stroma. During the third step, cells pass through the circulation and finally stabilize as a secondary tumor [1]. In parallel with this process, cancer stem cells cause variations in genetic aberrations of the primary tumor, resulting in the formation of secondary tumors with different properties. Such altered secondary tumors can be resistant to classical anticancer therapy [2,3]. Josić et al. emphasized that in the process of developing strategies for the diagnosis and treatment of tumors, particular attention should be paid to the glycosylation processes. Since tumor development is a consequence of gene expression alterations and alterations in protein expression, tumor-related glycosylation alterations could be also new possible targets for diagnosis and therapy [4].

Due to the complexity of metastasis development, treatments for such condition should be complex and target not only a single target gene [1]. Nowadays, therapies for cancer treatment are focused on the combination of several mono-targeting drugs or the development of a single drug with a few targets. Ideas for the development of drugs with such properties can be found in plant-derived agents that show multi-targeting potential. Usually, plant-derived drugs are, as compared to synthetically developed drugs, easily available, less expensive, and safer.

The field of nutrigenomics studies the effects of various dietary components on gene expression and molecular mechanisms via “omics” technologies. Nutraceuticals comprise any food or individual food components that provide any form of health benefits. Nowadays, there is a lot of evidence how different nutraceuticals, especially those with polyphenolic-based structures, possess anticancer properties. However, since metastases are responsible for the majority of cancer deaths, focus should be also put on the influence of these constituents on this step in cancer development and progression. The greatest challenge in metastasis treatment with nutraceuticals is in clarifying the genomic, proteomic, and metabolomic pathways of metastasis, as well as understanding the nutritional role in cancer cell communication. The complexity of mechanisms of nutrient activities makes this field of research difficult to investigate and define. There is a clear absence of understanding of the connection between consumption and genomic, proteomic, and metabolomic effects. It is well known that nutrients affect several different cell pathways, and their anticancer and anti-metastatic effects are systematic and often a result of a synergistic effect of several different constituents. Additionally, consumption of such constituents through everyday food will probably not have the desired effect on metastasis inhibition, due a failure in achieving the concentrations required for the desired effect. Moreover, a diverse range of research designs and the usage of different cell types and different complexes of various dietary constituents make the comparison of existing results almost impossible.

Each individual has a different microbiome that results in different intestinal absorption and processing of phytochemicals. Therefore, personalized nutrition is crucial. The diet should be adjusted to each person, since there are over three million genetic variations between two random persons. Diet can cause epigenetic alterations, which can have various effects on the genomic level of different individuals. If we consider diet as a factor that maintains metabolic balance and keeps the body in homeostasis, it is clear that each individual will need a different diet depending on his genome, proteome, and metabolome.

The connection between cancer and human diet has been extensively documented in past few years, but diet adjustment in order to prevent and/or treat developed metastasis is lacking. Therefore, in this review we focus on results published in the past five years on the link between nutrigenomics and metastases.

## 2. The Role of Nutrigenomics in Metastasis Proliferation

Nowadays, most information is publicly available. A large number of traditional natural drugs are available, very often without a necessary prescription from a physician. Due to this, self-treatment with complementary methods that often include consumption of easily available plant-based extracts and its constituents is on the rise.

Despite the advantages of such remedies on overall health, some of them can have adverse effects in the late stages of some diseases. Emerging research has shown that some plant-based drugs can negatively impact late-stage cancer. Indeed, consumption of some antioxidants, phytoestrogens, etc., can negatively influence prognosis of patients with developed cancer via promotion of metastasis development and proliferation. In the next section, we focus on the latest studies showing a negative influence of nutraceuticals on the development of metastases.

### 2.1. The Enhancing Effects of Specific Dietary Compounds on Metastasis Development

#### 2.1.1. Targeting Reactive Oxygen Species (ROS)

**Figure 2:**
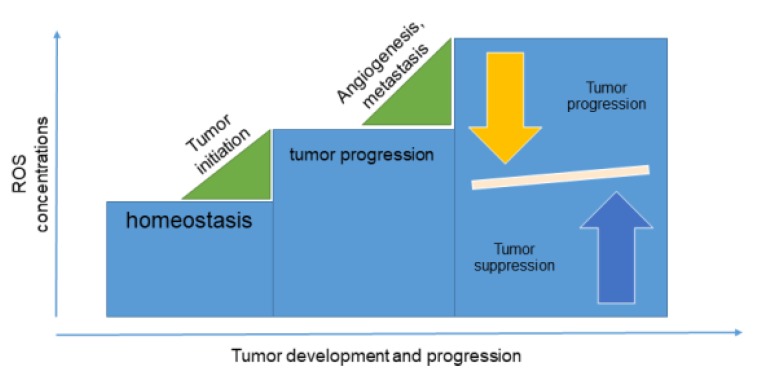
Reactive oxygen species (ROS) in tumor development.

Antioxidants have been extensively analyzed as a natural solution for cancer treatment. The main idea is that accumulation of reactive oxygen species (ROS) in the organism causes cellular damage in human body that often leads to cancer development. ROS are crucial for homeostasis and, at lower concentrations, regulate intracellular processes. However, overproduction of ROS, which is usually the consequence of the both endogenous and exogenous sources such as pollution, drugs, smoking, unhealthy lifestyle, etc., causes oxidative stress, which may initiate carcinogenesis (Figure 2). ROS are involved in carcinogenesis on several levels; they initiate cell transformation, enable tumor survival, promote tumor proliferation and invasion, and induce tumor cell angiogenesis and metastasis [5]. Accordingly, it would be expected that a diet including antioxidants would have beneficial effects on the health of a patient with cancer [6]. However, cancer cells are more sensitive to ROS than normal cells [7], suggesting that elevated ROS concentrations are more harmful to the cancer than to the human body. These findings were confirmed by Harris et al., where authors revealed that malignant cells used two pathways for initiation and progression: glutathione and thioredoxin antioxidant pathways [8]. Inhibition of these pathways caused cancer cell death, suggesting that disabling the antioxidant synthesis can be chemopreventive. Additional confirmation of these findings came from Piskounova et al., who revealed that antioxidants could promote production of distant metastases in melanoma cells [9]. However, it was shown that tumor cells could undergo metabolic changes to adapt to high levels of ROS and therefore successfully develop metastases.

In their research, Gal et al. showed that specific antioxidants could increase melanoma metastases incidence in mice [10]. The research was inspired by previous findings, which showed that human lung cancer proliferation and tumor growth was increased in conditions with low amounts of ROS. Considering the fact that phytochemicals largely possess antioxidant activity, it was concluded that consumption of phytochemicals with such properties would positively influence tumor development. Due to these findings, authors decided to analyze the influence of antioxidant consumption on a cancer sensitive to changes in redox status—malignant melanoma. Research was conducted in vivo on mouse models. The results revealed that administration of two antioxidants with completely different physical and chemical properties, N-acetylcisteine (NAC) and vitamin E, increased tumor migration and invasiveness. Additionally, NAC also increased lymph node metastases. Interestingly, the number and size of primary tumors were not affected at all, and nor was the degree of the proliferation. The negative results of antioxidant usage for metastasis treatment can be easily explained by the fact that tumor cells that leave the primary tumor experience oxidative stress, and every compound that can lower the amount of ROS in cells can promote tumor progression. However, the authors stressed that these results were specific to the antioxidants, cell lines, and mouse models used in their study, confirming the need for further research with the same experimental conditions as well as clinical trials [10].

#### 2.1.2. Targeting Estrogen Receptors

Breast cancer is one of the most invasive cancer types, and often metastasizes to the bones [11]. This disease can be divided into estrogen receptor (ER)-positive or (ER)-negative breast cancer and further into several subtypes depending on the presence of other biomarkers such as human epidermal growth factor receptor 2 (HER2) or progesterone receptors. The presence of all these cancer types makes it impossible to develop a unique anticancer drug, and sets the need for individualized treatments. Recent studies have revealed that this tumor can develop micro-tumors in the bone marrow and that this condition is a sign of poor prognosis for patients [12]. Unfortunately, breast cancer metastases develop before they can be detected routinely. Due to these challenges, researchers are focused on diet modulation as an effective preventing strategy for breast cancer, but also as a therapy for already developed metastases.

Many studies on the influence of soy isoflavones on breast cancer showed that soy consumption lowered cancer recurrence [13,14,15]. However, the effect of soy consumption on cancer development is not clear. Taking into consideration that there are two major groups of breast cancers, and also that isoflavones mainly target estrogen receptors, it is obvious that these nutrients will not have the same effects on all breast cancers [16]. Additionally, the majority of studies claiming beneficial effects of soy consumption did not question the influence of these constituents on different cancer stages, i.e., metastasis development.

A study by Yang et al. provided new insight on the behavior of metastases that originate from breast cancer and revealed a negative impact of isoflavone consumption on further prognosis for patients [17]. Their first study conducted on mice showed that a diet high in isoflavones could enhance development of pre-existing estrogen-dependent breast tumors in a dose-dependent manner. In more detail, concentrations of isoflavone genistein greater than 150 ppm significantly increased cell proliferation and expression of pS2 in athymic mice implanted with estrogen-dependent tumors [18]. In their next study, the authors analyzed the influence of the mixture of isoflavones on breast cancer with bone micro-metastases using an 4T1 intratibial model established by injecting a small number of 4T1 cells into the tibia of female Balb/c mice. They discovered that the mixture of genistein, daidzen, and (–)-equol could enhance breast cancer growth and increase lung metastasis incidence [17]. These tumor- and metastasis-promoting results were obtained in vivo, where this isoflavone mixture (that was mixed with the AIN-93G powder to a concentration of 750 mg/kg and provided to mice at the amount of 5 g/day) increased not only the tumor growth in bone, but also lung metastasis from the 4T1 tumors in bone. However, when analyzed in vitro on cultured 4T1 cells, the isoflavone mixture was less potent in tumor and metastasis promotion, suggesting that there was probably a systemic effect between the host, tumor, and analyzed molecules.

#### 2.1.3. Targeting Other Cellular Pathways

In contrast to research mainly focused on the analysis of primary tumors or metastatic carcinomas in the last phase of metastases development, Wu et al. were focused on circulating tumor cells of breast cancer and examined the influence of different dietary factors on patients’ prognosis [19]. The authors investigated the influence of procyanidine, curcumin, β-sitosterol, tea polyphenols, and perilyl alcohol on circulating tumor cells, which are strong predictors of metastasis levels, in vivo. The experiment revealed smaller primary tumors in mice treated with the nutrients mentioned above. Additionally, the number of lung metastases was also lower after administering each compound (except for β-sitosterol), with procyanidine being the most potent in anti-metastatic activity. Similarly, procyanidine was the best at lowering the number of the circulating tumor cells, with an efficacy of around 75%. Interestingly, β-sitosterol was effective in lowering the volume of the primary tumor cells as well the number of the circulating tumor cells, but at the same time this constituent increased the number of lung metastases. The authors elucidated these findings with the theory that β-sitosterol is a pro-proliferative compound which probably causes an increase in circulating cancer stem cells, which have a higher metastatic potential than general circulating tumor cells. Additionally, the authors stressed the need to expand anti-cancer and anti-metastatic research on circulating cancer stem cells, as they may be more precise in estimating cancer metastasis development.

### 2.2. The Enhancing Effects of a High-Fat and/or High-Carbohydrate Diet on Metastasis Development

Obesity is one of the risk factors for cancer development. The main reason is that adipose tissue is considered an endocrine organ that produces its own proinflammatory cytokines, which can cause systematic inflammation. This inflammation can further trigger cancer occurrence and growth or stimulate the spread of metastases.

In addition to the analysis of influence of individual nutraceuticals on metastasis development, Wu et al. fed mice with high-carbohydrate and high-fat diets in order to determine if the two different diet types influenced the number of circulating tumor cells. The results revealed an increase of circulating tumor cells after administration of both diet types. However, only the high-carbohydrate diet induced enlargement of primary tumors [19]. Yan and DeMars examined the impact of high-fat diet on spontaneous metastasis of Lewis lung carcinoma. It was shown that this diet increased pulmonary metastasis in Lewis lung carcinoma by 60%, tumor cross-sectional area by 82%, and tumor volume by 130% in comparison to the control group. The low- (control) and high-fat diets had the same numbers of total calories, but the ratio of carbohydrates and fats was changed. Around 45% of total calories in the high-fat diet were provided from corn oil. The most significant factor for the negative influence of the high-fat diet was plasminogen activator 1, which is a serine protease inhibitor that is produced by the host and also by the adipose tissue [20].

The same approach based on a different model was used in a paper by Sundaram and Yan [21]. These authors analyzed the influence of a high-fat diet on MMTV-PyMT transgenic mice that were usually used to study luminal B breast cancer. The high-fat diet was achieved by replacing corn starch (the main component of the control-group diet) with soybean oil. The results were similar to those of the above-mentioned research: the high-fat diet increased primary tumor progression by 59%, primary tumor weight by 60%, and number of lung metastasis by 147%. These findings were attributed to enhanced concentrations of proinflammatory cytokines and up-regulation of angiogenic signaling.

Further research, which included a new approach, was conducted by Yan and Combs Jr. These authors analyzed the influence of high-fat diet on the inhibitory effect of methylseleninic acid on metastatic development of Lewis lung cancer [22]. The study was conducted on mice in which tumor was induced, and which were fed with low-fat or high-fat diet. Methylseleninic acid was used as an anti-metastatic agent as it was previously shown that it could reduce spontaneous metastasis of Lewis lung carcinoma [23]. The high-fat diet increased pulmonary metastases by 17% and reduced the positive anti-metastatic effect of methylseleninic acid in comparison with a low-fat diet [22]. The authors associated the negative results of high-fat diet with increased plasma concentrations of adipose-derived inflammatory cytokines. These findings showed that the activity of certain anti-metastatic agents was highly influenced by eating habits of each individual [22]. The approach used in this research is a valuable model for further analysis of the influence of dietary modifications on inhibitory effects of anti-metastatic agents. Furthermore, a higher level of personalized nutrition can be achieved by manipulation of ratios of major as well as minor food components. For example, in the context of fats, the authors suggest that further investigation should be focused on addressing the influence of different ratios of specific fatty acids on tumorigenesis. The major component of corn oil, which was used as a major source of fats in two mentioned studies [20,22], is linoleic fatty acid (57%), and it contains only 1% α-linoleic acid, which is a fatty acid which exhibits anti-tumorigenesis effects. Therefore, some of the unwanted effects may not only be a consequence of a high-fat diet, but also of an unfavorable ratio of dietary components. Therefore, additional effort should be invested into exploring the effects of each dietary component on the development of metastases.

All mentioned biologically active compounds that could promote metastasis development are summarized in the Table 1.

## 3. The Role of Nutrigenomics in Metastasis Prevention

There is much evidence on the positive effects of single compounds and combined nutraceuticals on cancer prevention and treatment. Advances with respect to revealing the pathways in cancer metabolism (reprogramed energy production cycles, changes in gene expression, increased production of specific metabolites etc.) will lead to the determination of targets for cancer treatment. Certain nutraceuticals have shown positive effects in modulation of cancer gene expression, regulation of induced inflammation, angiogenesis, and proliferation. However, as stressed before, it is not only important which nutraceutical is administrated, but also at which stage of cancer development. As shown in previous sections, some dietary constituents can have beneficial effects on cancer prevention, but at the same time can enhance its progression via promotion of metastasis development. Therefore, in the next section, the compounds with proven positive effects on metastasis treatment will be discussed. List of all nutraceuticals (single biologically active compounds or mixtures) that could be used in the prevention of metastasis development is summarized at the Table 2.

### 3.1. The Preventive Effects of Specific Dietary Compounds on Metastasis Development

In contrary to previous findings that nutraceuticals acted as promotional agents in breast metastasis development, Wang et al. showed that ellagic acid limited the development of breast cancer metastases [24]. Their research was conducted on MMTV-PyMT mice, which are suitable for analysis of luminal B breast cancer. Anti-metastatic activity of ellagic acid is inhibition of the ACTN4 gene, which is responsible for breast cancer stem cell self-renewal and their metastatic abilities, and consequently for poor survival of breast cancer patients. Inhibition of the ACTN4/β-catenin interaction with ellagic acid resulted in degradation of β-catenin proteasome, which disabled cell–cell adhesion for cancer cell migration. This research determined a new target for breast cancer treatment, which is especially valuable considering previous efforts in this field that were mainly focused on phytoestrogen-like phenolics, for which it was shown that mechanism of action can be controversial.

Resveratrol, a plant phenol usually found in red grapes, is known for its strong antioxidant potential. In contrast to the previously discussed negative roles of antioxidants, resveratrol showed beneficial effects in colorectal carcinoma metastasis treatment via other mechanisms, rather than ROS neutralization. Buhrmann et al. revealed that resveratrol could reduce cell viability of colorectal carcinoma cells via inhibition of focal adhesion by inhibition of adhesion kinase [25]. This compound was also potent in activation of caspase-3 and induction of apoptosis, as well as in suppression of tumor invasion and inhibition of the proliferation and β1-integrin expression. Importantly, resveratrol was potent in nuclear factor-κB (NF-κB)-dependent gene end-product inhibition, a pathway involved in tumor invasion, metastasis, and apoptosis.

Epigallocatechin-3-gallate (EGCG) from green tea is another polyphenol that was proven to exhibit antitumor and anti-metastatic activity. It was shown that EGCG could inhibit tumor invasion and angiogenesis, which are essential steps for tumor growth and metastasis [35]. Zhang et al. demonstrated that EGCG suppressed melanoma cell growth and metastasis by targeting tumor necrosis factor receptor-associated factor 6 (TRAF6) activity. TRAF6 is an adaptor protein that mediates protein–protein interactions and is overexpressed in melanoma [26]. Along with EGCG, green tea is also a source of other biologically active compounds that showed an inhibitory effect on tumor metastases. For example, rhamnogalacturonan-II-type polysaccharide, a compound isolated from pectinase digests of mature tea leaves, can stimulate the immune system by increasing activity of macrophages and natural killer (NK) cells [27].

Selenium is an essential nutrient with promising anticancer properties documented in many clinical and preclinical studies [28,36,37]. However, not all forms of selenium are effective in reducing cancer risk. In the Safety and Efficacy Large-scale Evaluation of COX-inhibiting Therapies (SELECT) trial, supplementation with selenomethionine did not prevent prostate cancer development [38]. Sundaram et al. examined the effect of supplementation with more promising selenium form—methylseleninic acid (MSeA). MSeA is a proximal precursor of methylated selenium metabolites that can have anticancer activity. Dietary selenium supplementation with MSeA in MMTV-PyMT male mice inhibited mammary tumorigenesis and reduced the number of metastases found in the lungs. The authors showed that the multitargeting mechanism of MSeA action includes downregulation of the urokinase plasminogen activator system, angiogenesis inhibition, and inflammation suppression [28].

### 3.2. The Preventive Effects of the Mixture of Nutraceuticals on Metastasis Development

Roomi et al. used a mixture of specially chosen nutraceutical in order to target every step in ovarian cancer progression [29]. The influence on ovarian cancer metastases was examined in vivo and in vitro using the human ovarian cancer line A-2780. Their nutrient mixture contained ascorbic acid, lysine, proline, green tea extract, and quercetin. With this mixture, they managed to suppress ovarian cancer metastases in the lungs. Interestingly, mice that were fed with 0.5% nutrient mixture as a supplementary diet did not develop tumors at all in 80% of cases, suggesting that this mixture had a preventive effect on cancer development. Additionally, this group of mice also did not develop metastases in the lungs due to inhibition of matrix metalloproteinase-9 expression. These in vitro activities were achieved with concentrations of 250 µg/mL. However, before any further application of this mixture in metastases treatment, serum levels should be determined after mixture administration. Moreover, this mixture should be tested on other cancer metabolic pathways since compounds with antioxidant activity can promote metastasis development.

Furthermore, Ko et al. revealed an inhibitory effect of polyphenol mixture of plant *Euphorbia supina*, a plant traditionally used in Korean folk medicine for treatment of numerous diseases, on metastatic breast cancer MDA-MB-231 cells [30]. In vitro results showed that this plant could inhibit matrix metalloproteinase 9 and lysyl oxidase production, both mediators of metastasis development in breast cancer cells. In addition, vascular cell adhesion molecule-1 (VCAM-1) expression was decreased, consequently lowering the potential for an extravasation of breast cancer cells. These findings revealed that plant extracts containing a mixture of different nutraceuticals could have a synergistically beneficial effect on cancer and metastasis treatment. However, finding the exact mechanisms of action and afterwards standardizing such products, remains a challenge. This research was conducted only in vitro and it is not known how these constituents combined together would act systematically.

Wheatgrass is another plant with potential therapeutic implications in cancer treatment due to the cumulative biological effects of polyphenols and other constituents present in the extract. Shakya et al. applied in vitro and in silico approach to examine anti-metastatic effect of methanol extract of wheatgrass (MEWG) [31]. The experiment revealed that MEWG inhibited metastasis and angiogenesis in human laryngeal squamous cell carcinoma (Hep-2), probably via inhibition of the upstream phosphatidylinositol 3-kinase/protein kinase B (PI3K/AKT) pathway and by decreasing vascular endothelial growth factor (VEGF), matrix metalloproteinases (MMPs), and inflammatory marker protein cyclooxygenase-2 (COX-2). Docking studies showed that certain MEWG components inhibited the activation of PI3K and AKT (PKB), which are kinases involved in cancer cell proliferation, cancer survival, metastasis development, and promotion of angiogenesis.

Another remarkable study was conducted by Chen et al. [32], and proved the anti-metastatic effect of black rice anthocyanins (BRACs) on HER2+ breast cancer cells in vitro. The results indicated that BRACs suppressed metastases in breast cancer cells via the RAS/RAF/MAPK cellular pathway. BRACs inhibited mRNA expression and activation of key components of the RAF/MAPK pathway and decreased interactions of HER2 with downstream signaling components, as well as interaction of MMP2 and MMP9 with their upstream regulators.

The positive effects of selenium supplementation on metastasis treatment has been discussed previously (Section 3.1). However, further research showed that mixtures of nutraceuticals could have even better anti-metastatic effect than the consumption of a single compound. A combined treatment consisting of selenium and lactic acid bacterium was even more efficient in enhancing immune responses in cancer-bearing hosts. In this nutraceutical combination, *Lactobacillus brevis* bacteria were enriched with selenium [33]. *L. brevis* bacteria can reduce selenium ions to elemental selenium nanoparticles (SeNPs) and deposit them in intracellular spaces, forming a new Se organic nutraceutical product. [39]. The experiment showed that, in mice with highly metastatic breast cancer, oral administration of SeNP-enriched *L. brevis* increased the level of cytokines IFN-γ and IL-17 and augmented NK cytotoxicity and delayed-type hypersensitivity (DHT) responses [33]. Therefore, this oral formulation could be a good candidate for cancer therapy. The potential of fermented bacteria in fighting tumors and their metastases was also shown by Aragón et al. In this study, milk fermented by *Lactobacillus casei* CRL 431 (FM) showed promising effects in inhibition of the growth of breast cancer and its metastases in mice models [34]. The administration of FM not only reduced tumor growth, but also extravasation of tumor cells, tumor vascularity, and lung metastases, probably via modulation of the immune response. The administration of FM affected the immune reaction in the metastatic area by improving antitumor response associated to CD8^+^, increasing the CD4^+^ lymphocyte number and decreasing the infiltration of macrophages in the lungs.

## 4. Conclusions

The connection between nutraceuticals and metastasis development is still not unambiguous. Both the undetermined tumor cell pathways and the undefined systematic activity of nutraceuticals leave much room for future research in this field. Nutraceuticals show a wide range of biological activities and are usually inexpensive, easily available, and have no harmful effects. However, recent studies have shown that not all of the nutraceuticals have beneficial effects on cancer metastases. For instance, antioxidants can promote cancer initiation and progression, and soy isoflavones, which have been widely used in the prevention and treatment of breast tumors, are now categorized as pro-metastatic agents. Therefore, in some cases metastases can be more efficiently treated with pro-oxidants and constituents that prevent metabolic adaptations of cancer cells to oxidative stress. In order to avoid undesirable effects of nutraceuticals on tumors, the effect of each compound or group of compounds should be tested at different stages of cancer development. The big challenge is the fact that one compound can have beneficial effects on reduction of a primary tumor, but afterwards can promote the spread of its metastases. This obstacle must be overcome before some nutraceuticals can be used in metastasis treatment.

## Figures and Tables

**Table 1:** List of biologically active compounds that could promote metastasis development.

Source	Compound	Type of the Experiment	Effect and Mechanism (if Analyzed)	Reference
NA*	N-acetylcisteine	In vivo: Mice with endogenous malignant melanoma	Increase in lymph node metastases Increase in migration and invasiveness of human melanoma cells by enrolment in the glutathione pathway	[10]
NA	Vitamin E		Increase in migration and invasiveness of human melanoma cells by enrolment in the glutathione pathway	
Soy	Genistein	In vivo: Breast cancer with bone metastases using a model of murine mammary cancer (4T1 cells)	Stimulation of the metastasis formation in lungs Increase of Ki-67 protein expression in metastasized tumors	[17]
	Daidzein			
	(-)-Equol			
	Mixture of a soy isoflavones			
	β-sitosterol	In vivo: Circulating tumor cell capture method using a mouse tumor model	Increase in lung metastases (potential increase in number of circulating cancer stem cells)	[19]
High-carbohydrate diet		In vivo: Circulating tumor cell capture method using a mouse tumor model	Enlargement of primary tumors Higher number of circulating tumor cells	[19]
High-fat diet			Higher number of circulating tumor cells	
High-fat diet		In vivo: Lewis lung carcinoma (spontaneous metastasis); PAI-1-deficient and wild type mice	Increase in the number of pulmonary metastases, tumor cross-section area, and tumor volume by increased expression of the plasminogen activator inhibitor-1	[20]
High-fat diet		In vivo: MMTV-PyMT mice; luminal B breast cancer	Enhanced primary tumorigenesis and metastasis by increasing proinflammation and angiogenesis signaling	[21]

*NA: not applicable.

**Table 2:** List of nutraceuticals (single biologically active compounds or mixtures) that could be used in the prevention of metastasis development.

Source	Group	Compound	Type of the Experiment	Effect and Mechanism (if Analyzed)	Reference
Chemical standard	Phenolic compound	Ellagic acid	In vivo: MMTV-PyMT mice; luminal B breast cancer	Inhibition of the actinin alpha 4 (ACTN4) gene (responsible for breast cancer stem cell self-renewal and their metastatic abilities)	[24]
Chemical standard		Resveratrol	In vitro: Colorectal cancer cell lines (HCT116 and SW480)	Inhibition of nuclear factor-κB (NF-κB)-dependent gene end-products	[25]
Chemical standard		Epigallocatechin-3-gallate (EGCG)	In vitro: Human malignant melanoma cell lines SK-MEL-5, SK-MEL-28, A375, G361, and HEK293T	Suppression of melanoma cell growth and metastasis by targeting tumor necrosis factor receptor associated factor 6 (TRAF6) activity	[26]
Mature tea leaves	Polysaccharide	Rhamnogalacturonan-II-type polysaccharide	In vivo: Mice with lung metastases	Stimulation of the immune system by increasing activity of macrophages and natural killer (NK) cells	[27]
			In vitro: Yac-1 tumor cells (Moloney murine leukemia virus-induced lymphoma cell line)		
Dietary supplement	Trace mineral	Methylseleninic acid (MSeA)	In vivo: MMTV-PyMT male mice; luminal B breast cancer	Multitargeting mechanism including downregulation of the urokinase plasminogen activator system, angiogenesis inhibition, and inflammation suppression	[28]
Nutrient mixture (EPQ)	Multiple active components	Mixture containing ascorbic acid, lysine, proline, green tea extract, and quercetin	In vivo: Mouse tumor model	Inhibition of matrix metalloproteinase (MMP) 9 expression	[29]
			In vitro: Human ovarian cancer line A-2780; ovarian cancer metastases		
*Euphorbia supina*	Phenolic compounds	Polyphenol mixture of plant *Euphorbia supina*	In vitro: Metastatic breast cancer MDA-MB-231 cells	Inhibition of MMP 9 and lysyl oxidase production	[30]
				Decrease of vascular cell adhesion molecule 1 (VCAM-1) expression	
Wheatgrass	Multiple active components (including phenolic compounds)	Methanol extract of wheatgrass (MEWG)	In vitro: Human laryngeal squamous cell carcinoma (Hep-2) In silico approach	Inhibition of the upstream PI3K/AKT pathway	[31]
				Reduction of vascular endothelial growth factor (VEGF), MMPs and inflammatory marker protein cyclooxygenase-2 (COX-2)	
Black rice	Phenolic compounds	Black rice anthocyanins (BRACs)	In vitro: HER2+ breast cancer cells	Inhibition of mRNA expression	[32]
				Activation of key components of the RAF/MAPK pathway	
				Decreased interactions of human epidermal growth factor receptor 2 (HER2) with downstream signaling components from the RAF/MAPK pathway	
				Decreased interaction of MMP2 and MMP9 with their upstream regulators	
Dietary supplement	Probiotic and trace mineral	*Lactobacillus brevis* bacteria enriched with selenium—elemental selenium nanoparticles (SeNPs)	In vivo: Metastatic form of mouse breast cancer	Increased cytokines IFN-γ and IL-17	[33]
				Increased NK cytotoxicity—delayed-type hypersensitivity (DHT) responses	
Fermented milk	Probiotic	Milk fermented by *Lactobacillus casei* CRL 431 (FM)	In vivo: Mouse breast cancer model	Modulation of the immune reaction in the metastatic area by improving the antitumor response associated to CD8^+^, increasing the CD4^+^ lymphocyte number, and decreasing the infiltration of macrophages in the lungs	[34]
